# Identification of differentially expressed genes and signaling pathways with *Candida* infection by bioinformatics analysis

**DOI:** 10.1186/s40001-022-00651-w

**Published:** 2022-03-21

**Authors:** Guo-Dong Zhu, Li-Min Xie, Jian-Wen Su, Xun-Jie Cao, Xin Yin, Ya-Ping Li, Yuan-Mei Gao, Xu-Guang Guo

**Affiliations:** 1 Department of Oncology, Guangzhou Geriatric Hospital, Guangzhou, 510180 China; 2 Department of Clinical Laboratory Medicine, The Third Affiliated Hospital of Guangzhou Medical University, Guangzhou, 510150 China; 3 Department of Pediatrics, The Pediatrics School of Guangzhou Medical University, Guangzhou, 510182 China; 4 Department of Clinical Medicine, The Second Clinical School of Guangzhou Medical University, Guangzhou, 511436 China; 5 Key Laboratory for Major Obstetric Diseases of Guangdong Province, The Third Affiliated Hospital of Guangzhou Medical University, Guangzhou, 510150 China

**Keywords:** *Candida*, High-throughput sequencing, Differentially expressed genes, Signaling pathways, Bioinformatics analysis

## Abstract

**Background:**

Opportunistic *Candida* species causes severe infections when the human immune system is weakened, leading to high mortality.

**Methods:**

In our study, bioinformatics analysis was used to study the high-throughput sequencing data of samples infected with four kinds of *Candida* species. And the hub genes were obtained by statistical analysis.

**Results:**

A total of 547, 422, 415 and 405 differentially expressed genes (DEGs) of *Candida albicans*, *Candida glabrata*, *Candida parapsilosis* and *Candida tropicalis* groups were obtained, respectively. A total of 216 DEGs were obtained after taking intersections of DEGs from the four groups. A protein–protein interaction (PPI) network was established using these 216 genes. The top 10 hub genes (FOSB, EGR1, JUNB, ATF3, EGR2, NR4A1, NR4A2, DUSP1, BTG2, and EGR3) were acquired through calculation by the *cytoHubba* plug-in in Cytoscape software. Validated by the sequencing data of peripheral blood, JUNB, ATF3 and EGR2 genes were  significant statistical significance.

**Conclusions:**

In conclusion, our study demonstrated the potential pathogenic genes in *Candida* species and their underlying mechanisms by bioinformatic analysis methods. Further, after statistical validation, JUNB, ATF3 and EGR2 genes were attained, which may be used as potential biomarkers with *Candida* species infection.

**Supplementary Information:**

The online version contains supplementary material available at 10.1186/s40001-022-00651-w.

## Background

*Candida* species colonizes human mucosal surfaces as commensals, which can turn to pathogenic behavior under certain conditions, especially in immunocompromised patients [1]. Opportunistic *Candida* species includes *Candida albicans* (C. *albicans*)*, Candida krusei* (C. *krusei*), *Candida tropicalis* (C. *tropicalis*)*, Candida glabrata* (C. *glabrata*)*,* and *Candida parapsilosis* (C. *parapsilosis*). Among them, *Candida albicans* is the major pathogen isolated [2, 3], accounting for about 50% of all candidiasis cases with a total mortality rate of 43% [4, 5]. However, in the last decade, the proportion of *Candida* has been changed, with a reduction of *Candida albicans* and an increase in *Candida parapsilosis*, *Candida tropicalis, and Candida glabrata *[2, 6]. Non-albicans *Candida* such as *Candida glabrata, Candida parapsilosis, and Candida tropicalis* have now been identified as frequent human pathogens [7]. The number of patients and the geographic location determined that the overall distribution of the species *Candida albicans* was more common in Australia, Japan, Korea, Hong Kong, and Malaysia, whereas *Candida tropicalis* is more common in Pakistan and India [2, 8].

Candidiasis is a common bloodstream infection in hospitals around the world, causing high morbidity and mortality [2, 9]. In recent years, the emergence of *Candida* resistant strains brought about the further risk of clinical infection [10]. The major virulence factors of these pathogens were the *Candida* peptide and the extracellular aspartic proteases of the *Candida* peptide family [11]. Despite the introduction of intensive care facilities and modern antifungal drugs, the results of progress in curing *Candida* infections over the past decades have been disappointing [10, 12]. At present, adjuvant immunotherapy can further reduce the morbidity and mortality caused by *Candida* infection [13]. Therefore, understanding how host defense pathways participate in candidiasis is crucial for determining new targets for immunotherapy.

Sequencing technology has been applied to find targets for immunotherapies. High-throughput sequencing has become an important method for studying genomics, epigenomics, and transcriptome [14]. At present, some studies applied high-throughput sequencing to the identification and characterization of clinical microbiology. And when clinical data are more complex and contains multiple species, this technology is more reliable than normal sequencing [15].

To study the differentially expressed genes (DEGs) and signaling pathways related to *Candida* infection, this study applied bioinformatics analysis to analyze the gene expression profiles of human whole blood infected by four common *Candida* species including *Candida albicans, Candida parapsilosis, Candida glabrata,* and *Candida tropicalis*.

## Methods

### Data sources

The Gene Expression Omnibus (GEO, http://www.ncbi.nlm.nih.gov/geo/) database, attached to the National Center for Biotechnology Information (NCBI), was used to store gene expression datasets, series, and platform records. The gene expression profiles of GSE114174, GSE114175, GSE114177, GSE114178, and GSE114179 provided by Philipp Kämmer from the GEO database were downloaded. These gene expression profiles were attained by high-throughput sequencing. The number of infection and control samples is shown in Table 1. As shown in Table 1, The number of whole blood sample infected with Candida albicans, Candida glabrata, Candida parapsilosis and Candida tropicalis are 15, 15, 15 and 15, respectively. As a control group, GSE114179 includes three whole blood sample. These whole blood samples were donated by German volunteers. Additionally, the sources of samples infected with different *Candida* species were from Germany, America and Netherlands. Further details of the data sources from the GEO database for this study are shown in Table 1.

**Table 1:** **Details of the data sources from Gene Expression Omnibus (GEO) for this study**

GEO series (GSE)	Sample collection	Sample size	Infection vs control	GEO sample (GSM)	Sample source	GEO platform (GPL)
GSE114174	Homo sapiens whole blood infected with *Candida albicans*	18	15 vs 3	GSM3136879 ~ 96	Germany	GPL24974 Illumina HiSeq 2500 (*Candida albicans*; Homo sapiens)
GSE114175	Homo sapiens whole blood infected with *Candida glabrata*	18	15 vs 3	GSM3136897 ~ 99 GSM3136900 ~ 14	Germany	GPL24975 Illumina HiSeq 2500 ([*Candida*] *glabrata*; Homo sapiens)
GSE114177	Homo sapiens whole blood infected with *Candida parapsilosis*	18	15 vs 3	GSM3136915 ~ 32	Germany	GPL24976 Illumina HiSeq 2500 (*Candida parapsilosis*; Homo sapiens)
GSE114178	Homo sapiens whole blood infected with *Candida tropicalis*	18	15 vs 3	GSM3136933 ~ 50	Germany	GPL24977 Illumina HiSeq 2500 (*Candida tropicalis*; Homo sapiens)
GSE114179	Homo sapiens whole blood	3	0 vs 3	GSM3136951 ~ 53	Germany	GPL16791 Illumina HiSeq 2500 (Homo sapiens)
GSE42630	Homo sapiens PBMCs stimulated with *Candida albicans*	10	5 vs 5	GSM1046846 ~ 55	America & Netherlands	GPL16288 AB 5500xl Genetic Analyzer (Homo sapiens)

*PBMCs* peripheral blood mononuclear cells

### Data possessing and identification of DEGs

With R software, the data of GSE114174, GSE114175, GSE114177, GSE114178 and GSE114179 were batch-corrected and standardized using the *affy* software package, and the screening and identification of DEGs were carried out using the *limma* software package. Additionally, the *ggplot2* package was applied to draw a volcano map, whereas the *pheatmap* package was employed to make a heatmap to visualize the DEGs. Using *P* < 0.05 and |log2FC|≥ 1 as critical values, the gene expression profiles in infected samples and uninfected samples were compared.

### Gene Ontology (GO) and Kyoto Encyclopedia of Genes and Genomes (KEGG) analysis of DEGs

Gene Ontology was the main bioinformatics means used to annotate genes and analyze their biological processes. It includes the biological process (BP), molecular function (MF), and cellular component (CC), which reflects the conceptual category of gene product function, the biological processes of DNA metabolism and known differences between different organisms [16]. The KEGG is a database that can be used to understand biological systems and advanced functions from large-scale sequencing data generated by high-throughput sequencing technology [17]. In order to illustrate the biological function of genes and signal pathways involved in the vivo and cells, differentially expressed genes were annotated based on GO and KEGG analysis. The clusterProfler package in R software was used to performed the two analyses.

### Construction of protein–protein interaction (PPI) network and identification of hub genes

The DEGs of four transcription profile data (GSE114174, GSE114175, GSE114177 and GSE114178) were overlapped by using online tools (https://bioinfogp.cnb.csic.es/tools/venny/index.html). The construction of the PPI network was carried out using the Search Tool for the Retrieval of Interacting Genes (STRING) database (http://string-db.org/), which aimed at providing important estimates and integrations of protein–protein interactions, including functional and physical association [18]. Moreover, the *cytoHubba* plug-in in Cytoscape 3.7.2 software was applied to obtain the top 10 hub genes which ranked by Maximal Clique Centrality (MCC).

### Verification of intersection hub genes and construction of intersection gene–miRNA interaction

After identifying the intersection hub genes from four groups of data, the data of GSE42630 (https://www.ncbi.nlm.nih.gov/geo/query/acc.cgi?acc=GSE42630) obtained after the bioinformatics analysis were used for verification. GraphPad Prism 8.0 software was used for statistical analysis. The Gene–miRNA interaction was constructed using the miRTarBase v8.0 database [19] by Network Analyst (https://www.networkanalyst.ca/NetworkAnalyst/home.xhtml).

## Results

### Identification of DEGs associated with *Candida* infection

**Fig. 1:**
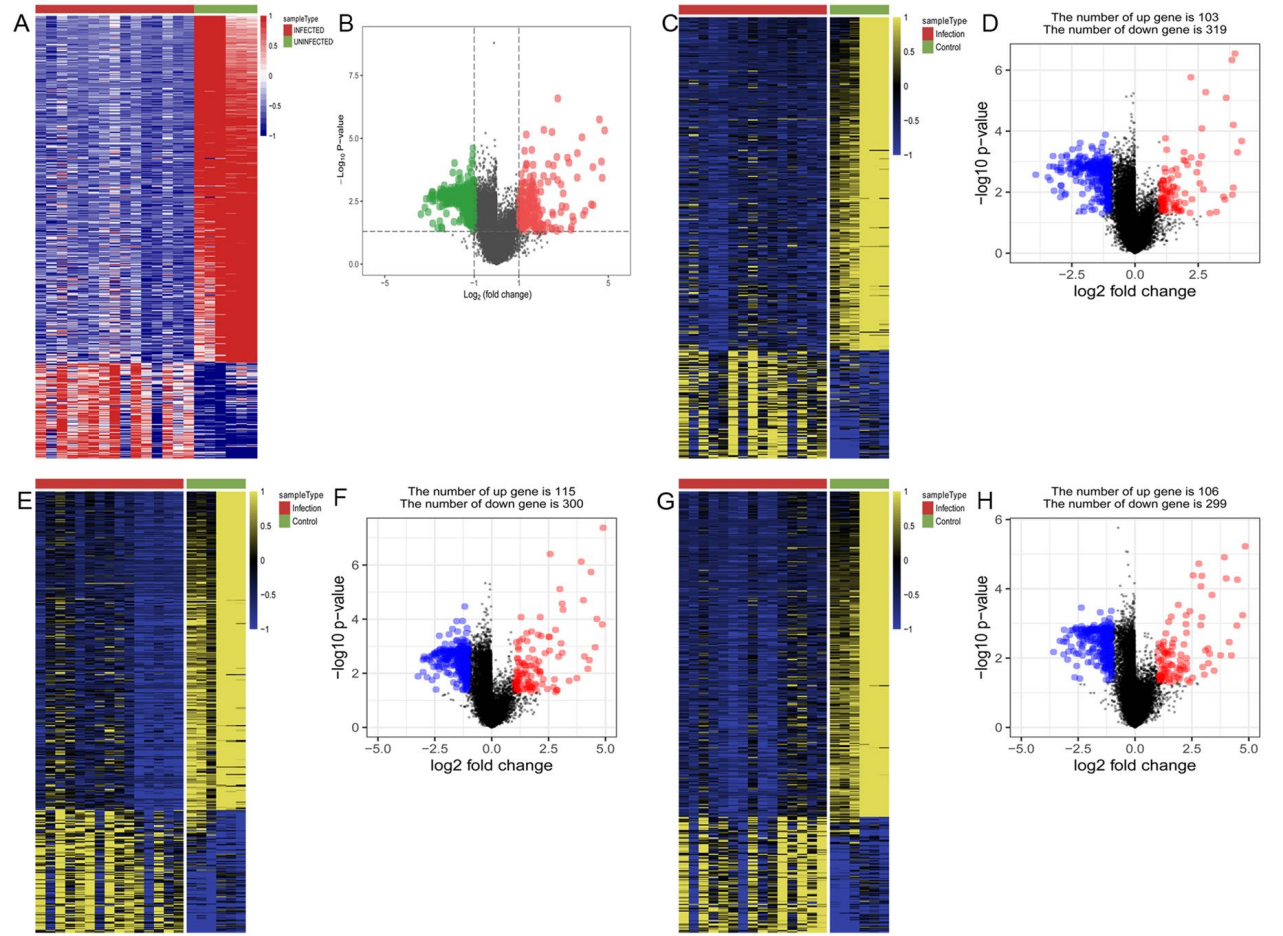
**Heat map and volcano map of DEGs from four groups of** ***Candida*** **species.** **AB** *Candida albicans*, **CD** *Candida glabrata*, **EF** *Candida parapsilosis*, and **GH** *Candida tropicalis*. In heat maps, gene expression data are converted into a data matrix. Each column represents the genetic data of a sample, and each row represents a gene. The color of each cell represents the expression level, and there are references to expression levels in different colors in the upper right corner of the figure. In volcano maps, red dots indicated up-regulated genes. The green or blue dots indicated down-regulated genes. Black dots indicated the rest of the genes with no significant expression change. The threshold was set as followed: *P* < 0.05 and |log2FC|≥ 1. FC: fold change

A total of 547, 422, 415 and 405 DEGs of *Candida albicans, Candida glabrata, Candida parapsilosis* and *Candida tropicalis* groups were separately obtained. Table 2 shows the detailed results of DEGs between the infection and control groups. The heat map of the DEGs proved that these DEGs could clearly distinguish between control samples and samples infected by C. *albicans* (Fig. 1B)*, Candida glabrata* (Fig. 1D)*, Candida parapsilosis* (Fig. 1F) and *Candida tropicalis* (Fig. 1H)*,* respectively. Figure 1A, C, E and G, respectively, shows the volcano plot of the DEGs for C. *albicans*, C. *glabrata*, C. *parapsilosis* and C. *tropicalis*.

**Table 2:** **Detailed results of differentially expressed genes (DEGs) between infection and control group**

Infection	Control	Infected with *Candida* species	Totally DEGs	Up-regulated DEGs	Down-regulated DEGs
GSE114174	GSE114179	*Candida albicans*	547	118	429
GSE114175	GSE114179	*Candida glabrata*	422	103	319
GSE114177	GSE114179	*Candida parapsilosis*	415	115	300
GSE114178	GSE114179	*Candida tropicalis*	405	106	299

### Go enrichment analysis of DEGs associated with *Candida* infection

**Fig. 2:**
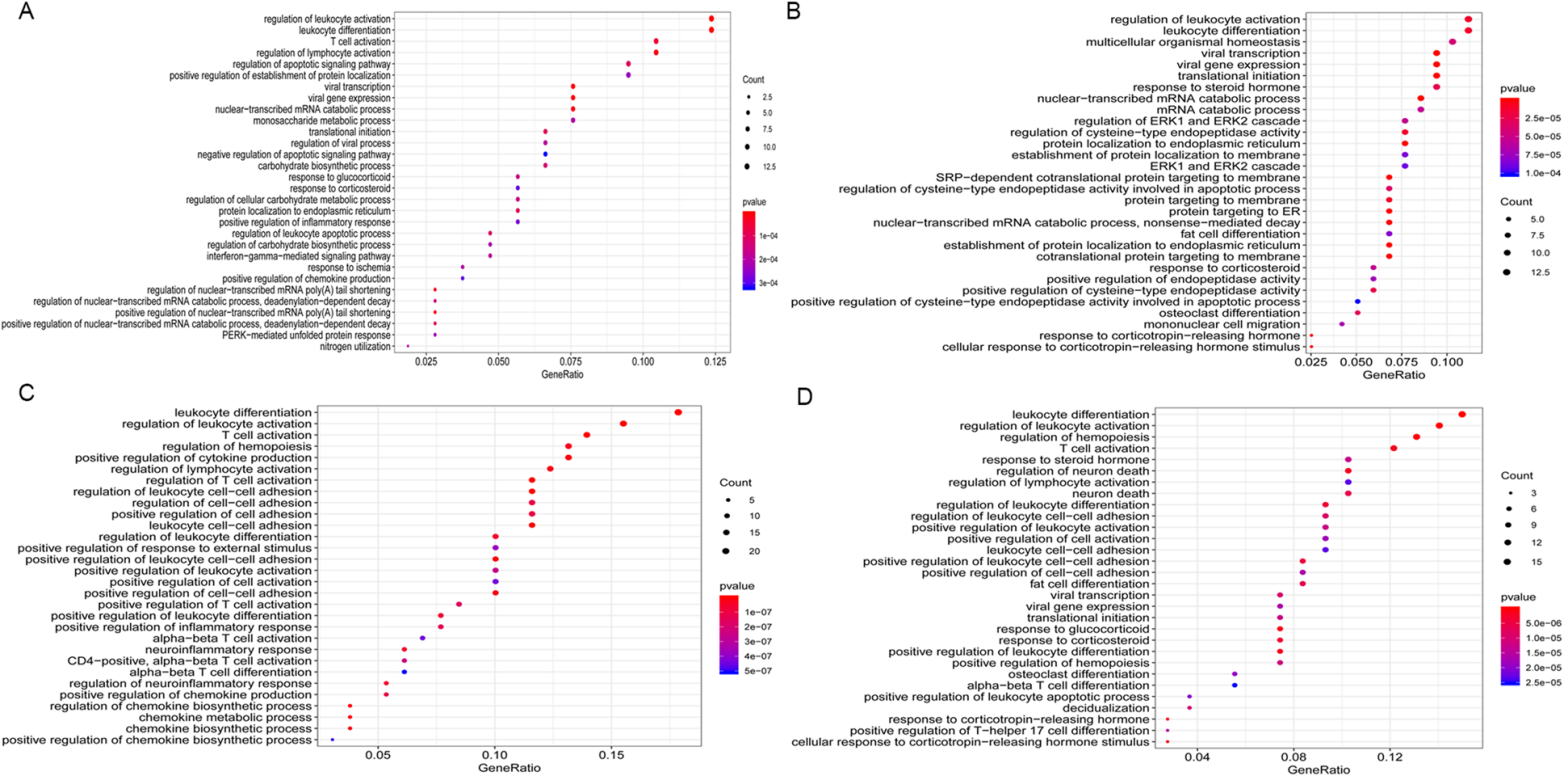
**The biological processes (BP) of GO analysis from four groups of** ***Candida*** **species.** **A** *Candida albicans*, **B** *Candida glabrata*, **C** *Candida parapsilosis*, and **D** *Candida tropicalis*

**Fig. 3:**
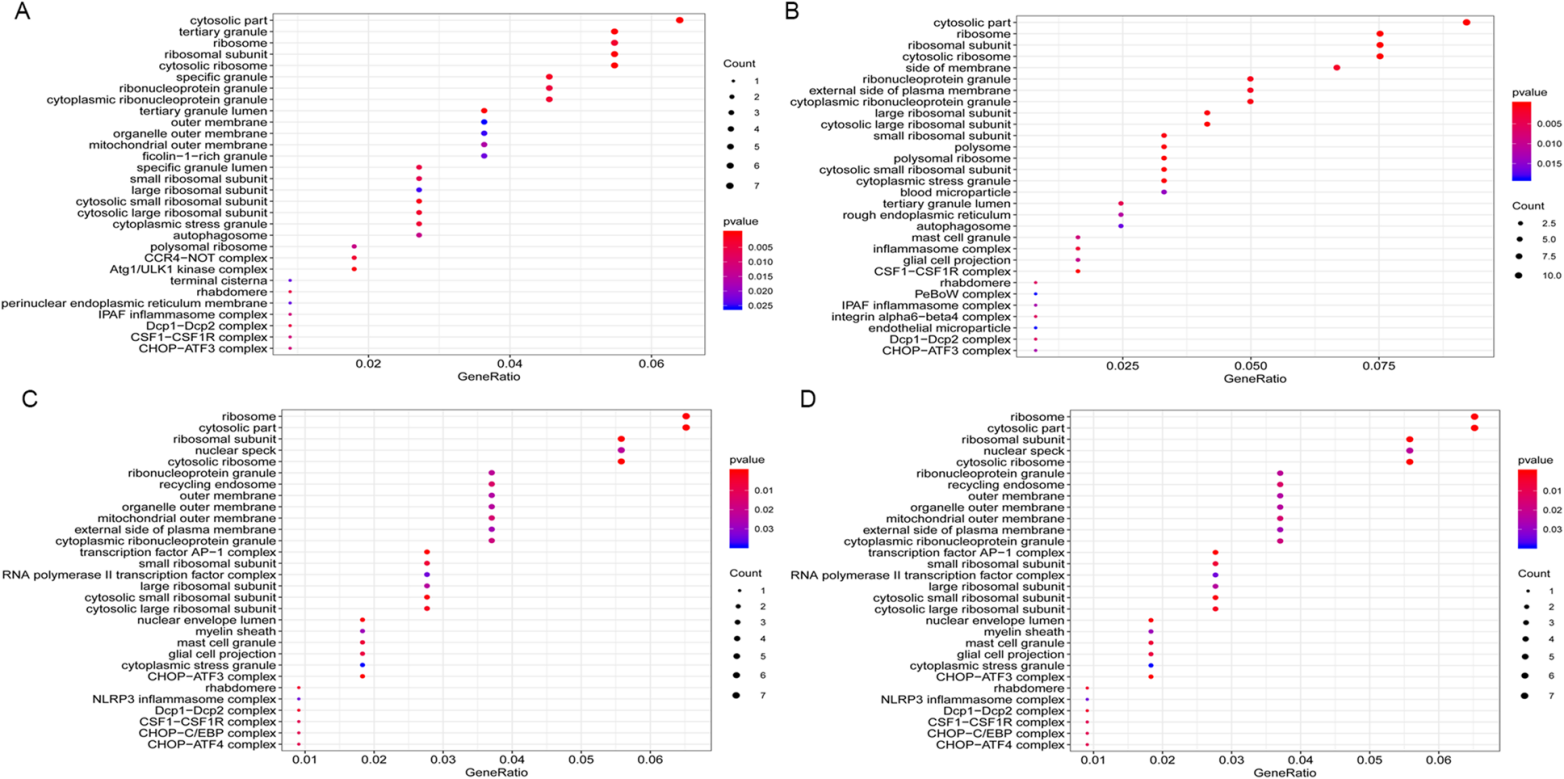
**The cellular components (CC) of GO analysis from four groups of** ***Candida*** **species.** **A** *Candida albicans*, **B** *Candida glabrata*, **C** *Candida parapsilosis*, and **D** *Candida tropicalis*

**Fig. 4:**
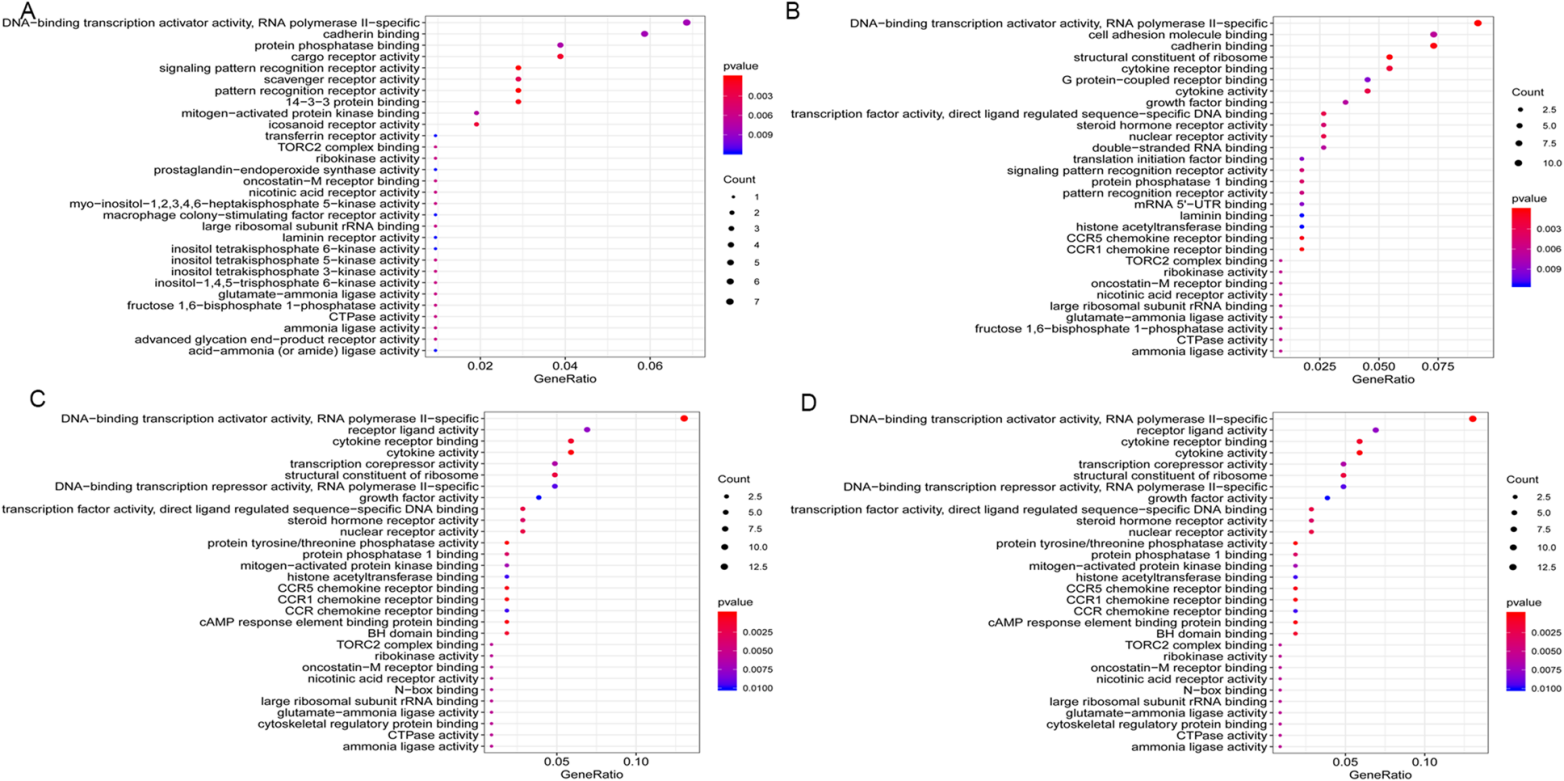
**The molecular function (MF) of GO analysis from four groups of** ***Candida*** **species.** **A** *Candida albicans*, **B** *Candida glabrata*, **C** *Candida parapsilosis*, and **D** *Candida tropicalis*

Figures 2, 3 and 4 represent the results of biological processes, cellular components and molecular function in the GO enrichment analysis. For the biological process, both the *C. tropicalis* and *C. parapsilosis* groups were significantly enriched in T cell activation, whereas the *C. albicans, C. tropicalis* and *C. parapsilosis* groups were significantly enriched in leukocyte differentiation and regulation of leukocyte activation. With regard to the cellular component, the *C. albicans, C. glabrata, C. parapsilosis* and *C. tropicalis* groups were significantly enriched in the cytosolic ribosome and cytosolic part. Regarding the molecular function, both *C. tropicalis* and *C. parapsilosis* groups were significantly enriched in cytokine activity and DNA-binding transcription activator activity, RNA polymerase II-specific. The top 5 terms of significant enrichment of GO analysis for *Candida albicans*, *Candida glabrata*, *Candida parapsilosis* and *Candida tropicalis* in the Additional files 1, 2, 3, 4 (Tables S1–S4).

### KEGG pathway enrichment analysis of DEGs associated with *Candida* infection

**Fig. 5:**
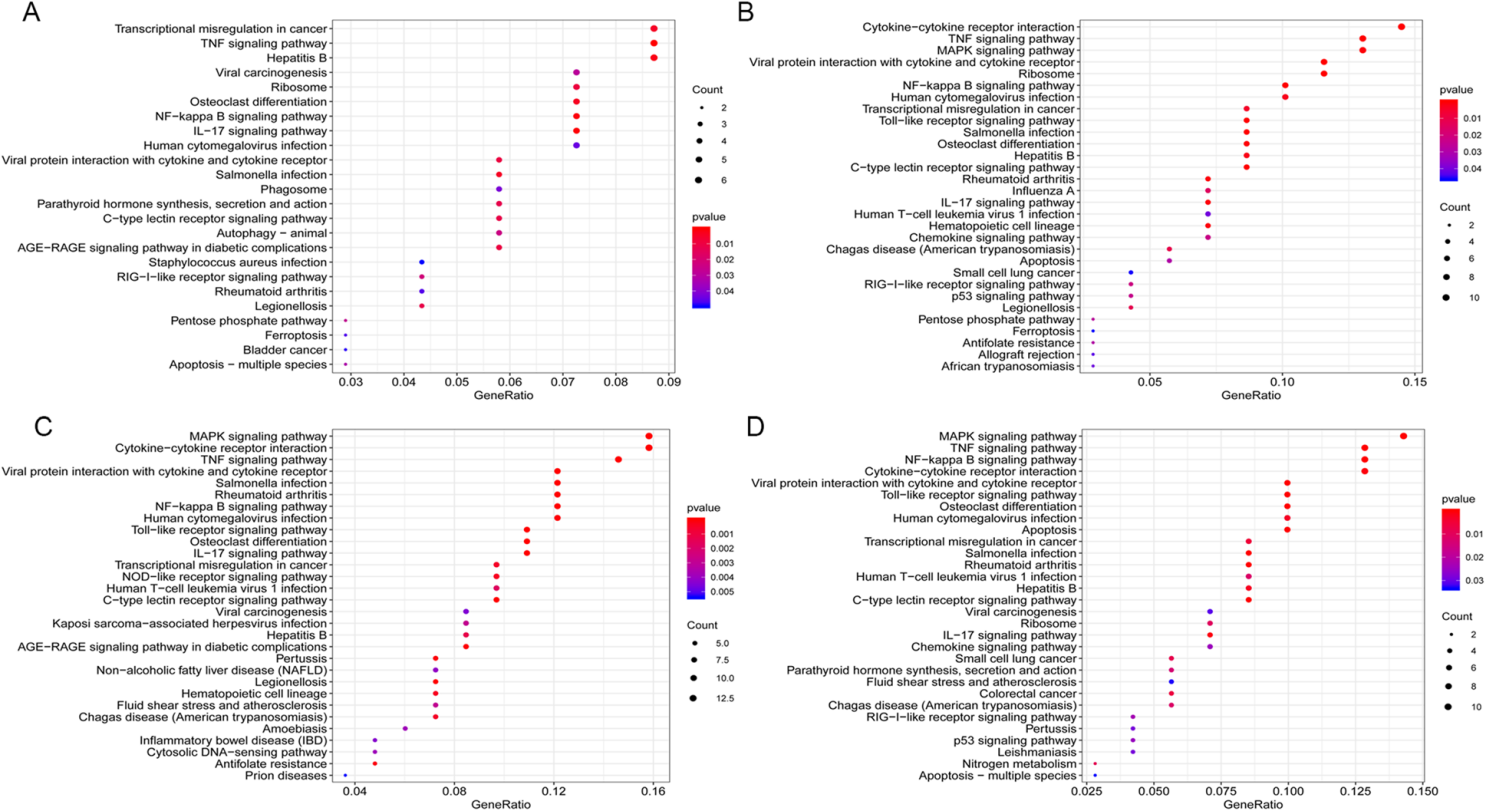
**The analysis of KEGG pathways from four groups of** ***Candida*** **species.**  **A** *Candida albicans*, **B** *Candida glabrata*, **C** *Candida parapsilosis*, and **D** *Candida tropicalis*

KEGG pathway analysis was conducted to identify the biological functions of DEGs. The results of the analysis are shown in Fig. 5. Five significant enrichment pathways were found simultaneously in *C. albicans, C. glabrata, C. parapsilosis* and *C. tropicalis*, which included the NF-kappa B signaling pathway, TNF signaling pathway, viral protein interaction with cytokine and cytokine receptor, salmonella infection and osteoclast differentiation. Additionally, the Toll-like receptor signaling pathway and MAPK signaling pathway were found simultaneously in *C. glabrata, C. parapsilosis* and *C. tropicalis*. The top 10 terms of significantly enriched KEGG pathways for *Candida albicans*, *Candida glabrata*, *Candida parapsilosis* and *Candida tropicalis* in the Additional files 5, 6, 7, 8 (Tables S5–S8).

### Construction of PPI network and hub genes identification

**Fig. 6:**
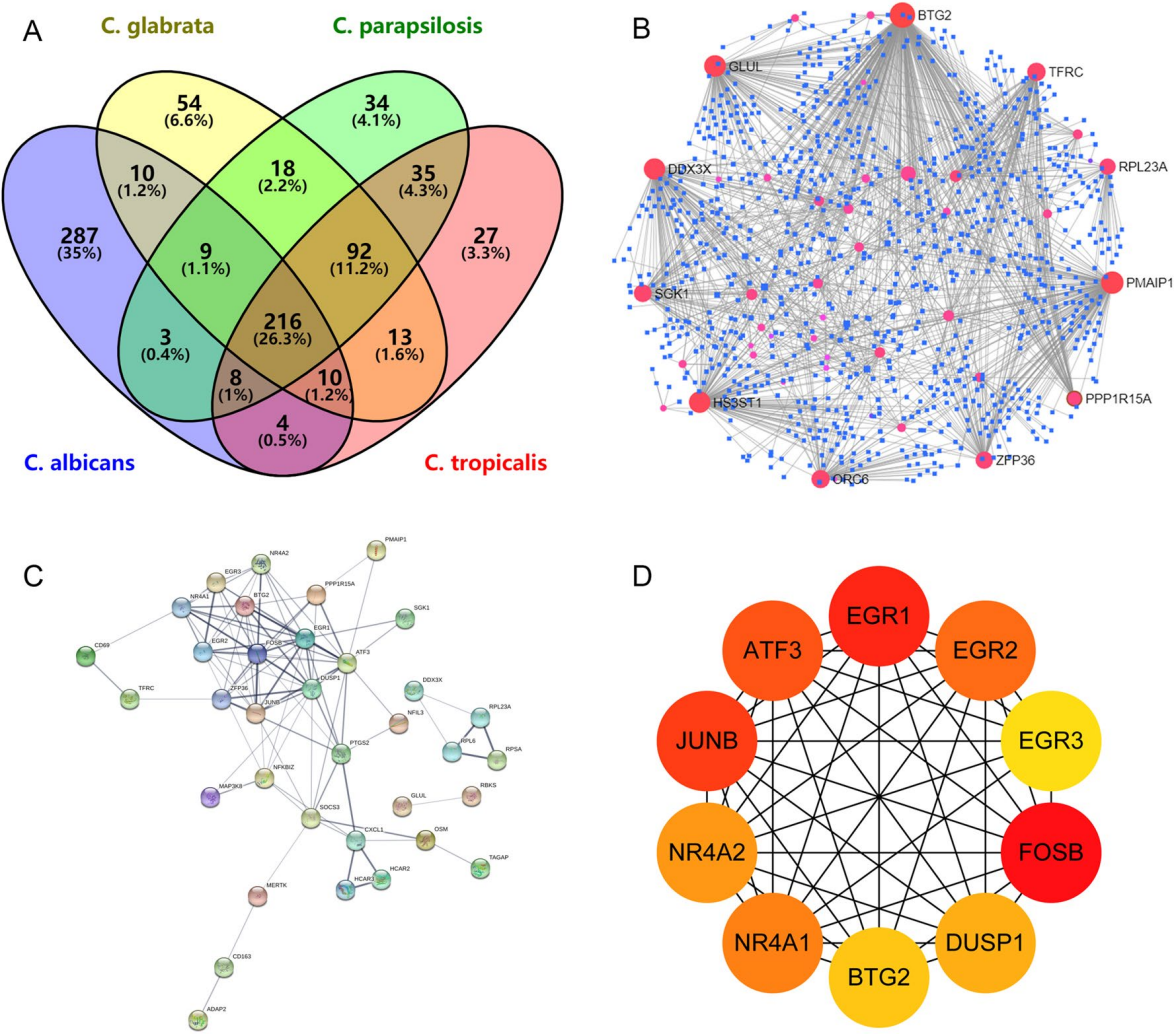
**The intersection results and relevant analysis of DEGs from four groups of** ***Candida*** **species.** **A** The overlapping results of DEGs of four groups of *Candida* species. Crossed regions indicate co-expressed DEGs. **B** The gene-miRNA network of overlapping DEGs based on miRTarBase v8.0 database. **C** The PPI network of overlapping DEGs. **D** The top 10 hub genes of PPI network

The overlapping results of DEGs of four groups of *Candida* species are shown in Fig. 6A. The PPI network and the top 10 hub genes of the *C. albicans*, *C. glabrata*, *C. parapsilosis*, and *C. tropicalis* groups are, respectively, shown in Additional files 9, 10 (Figs. S1 and S2). A total of 216 DEGs were obtained after taking the intersection of the DEGs from the four groups. The 216 genes were uploaded to STRING to construct a PPI network, consisting of 51 nodes and 96 edges (Fig. 6C). The top 10 hub genes obtained included FOSB, EGR1, JUNB, ATF3, EGR2, NR4A1, NR4A2, DUSP1, BTG2, and EGR3 (Fig. 6D).

### Construction of intersection gene and miRNA interaction and verification of intersection hub genes

**Fig. 7:**
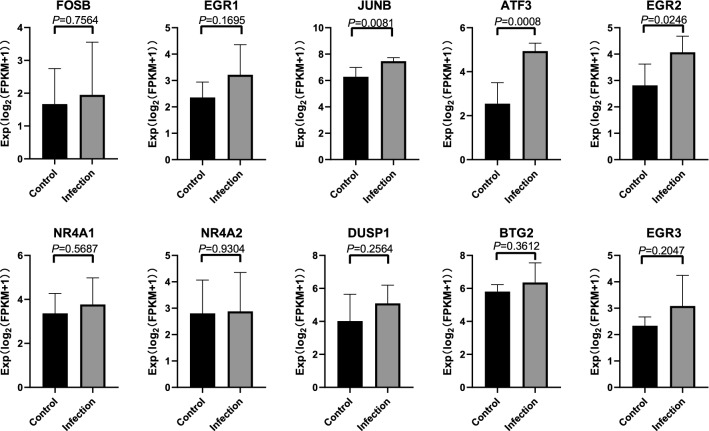
**Verification of hub genes associated with** ***Candida*** **infection.** *P*-value < 0.05 is considered to be statistically significant

The Gene-miRNA interaction of intersection genes is shown in Figure 6B. According to Degree, the top 10 genes interacted with miRNA were: BTG2, PMAIP1, GLUL, HS3ST1, DDX3X, TFRC, ORC6, SGK1, ZFP36 and RPL23A. In consideration of the rigorousness of this study, the data from the GSE42630 were used to verify the 10 intersection hub genes obtained. With *P* < 0.05 as the standard, the analytic results of the genes (JUNB, ATF3, EGR2) were found to be statistically significant (Fig. 7).

## Discussion

*Candida* species is one of the most common pathogens of invasive fungal infections among hospitalized patients [20]. Bioinformatics analysis can quantitatively analyze the gene expression of *Candida* species and identify the differentially expressed genes generated in cells, tissues or organisms that were infected with *Candida* species and uninfected. It is extremely important to understand the molecular mechanism of genetic regulation of *Candida* species and to better treat and diagnose diseases [21]. We downloaded the gene expression profiles relevant to the *Candida* infection from the GEO database. The obtained DEGs were used to perform GO enrichment analysis and KEGG pathway analysis, construct the PPI network, and gene–miRNA interaction network and identify the top 10 hub genes.

The GO enrichment analysis indicated that an increase in pattern recognition receptor activity may enhance the host’s defense against *Candida* species. The study has shown that when infected with *Candida* species, the first step to develop an immune response to *Candida* species is the recognition of invasive fungi [22]. At present, studies have found that PRRs of *Candida albicans* include TLRs, CLRs, NLRs and RIG-I-like receptors (RLRs) [22, 23]. Additionally, in our study, the leukocyte differentiation, regulation of leukocyte activation and T cells activation increased resistance to the invasion of *Candida* species, which was consistent with previous studies [24–26]. In addition to killing *Candida* by the production of reactive oxygen species (ROS) and phagocytosis, activated neutrophils can also release neutrophil extracellular traps (NETs), that capture *Candida* conidia and hyphae and contain the antimicrobial proteins to inhibit fungal growth [27, 28]. TH17 cell responses played an important role in mucosal host defenses against *Candida* by producing IL-17 and IL-22. These cytokines recruit and activate neutrophils, activate epithelial cells and release antifungal β-defensins cooperatively [29]. TH1 cell responses and IFNγ productions were quite important for the fungicidal activities of both neutrophils and macrophages [30].

In the current study, certain cellular signaling pathways identified using KEGG analysis were closely associated with *Candida* fungal infections. In addition to recognizing a wide variety of microbial products including lipoproteins, flagellin, lipopolysaccharides and bacterial DNA, signal transduction through TLRs also led to the production of inflammatory mediators [31]. Previous studies have shown that TLR4 mediated the induction of pro-inflammatory cytokines after *Candida* stimulation, whereas TLR2’s recognition of *Candida* primarily led to the release of anti-inflammatory cytokines [32]. TLRs initiate downstream signaling that culminates in the activation of nuclear factor kappa B (NF-κB), mitogen activating protein (MAP) kinases, or Interferon regulatory factors (IRFs), to regulate the expression of type I IFNs, cytokines and chemokines that ultimately should protect the host from infection by pathogens [33]. Primarily produced by activated macrophages, TNF signals are transmitted through two different cell surface receptors, TNF-R1 and TNF-R2 [34]. A number of experimental studies have revealed that the TNF-R1 activates most of the biological activity of TNF. The binding of TNF to TNF-R1 initiates downstream signaling that culminates in the activation of NF-kB and c-Jun, two major transcription factors [35, 36]. The NF-kappa B pathway is divided into two different but interacting pathways: the classical NF-kappa B essential modulator (NEMO)—a dependent pathway and the alternate NEMO—an independent pathway. While the classical NF-κB signaling pathway, induced by TNF-α, IL-1, or by-products of bacterial and viral infections, is mainly associated with inflammatory, proliferative, and survival responses, the activation of the noncanonical pathway results in a chemokine expression. Taken together, detecting these pathways may be helpful to predict the progression of Candidiasis [37].

Protein–protein interactions and acquired networks are very important in most biological functions and processes, as most proteins seem to activate their functions through interactions [38]. The hub genes screened through the PPI network are closely related to the potential molecular mechanism of *Candida* infection in humans. Therefore, a total of 10 hub genes were selected in this study. Keeping in mind the rigor of this study, the data of GSE42630 was used to verify the 10 hub genes. Through verification, among the 10 hub genes, the three hub genes—JUNB, ATF3, and EGR2—were significant statistical significance. Studies have shown that JUNB possesses an important effect during the growth of Treg cells, as it promotes IL-2 signal transduction [39]. Therefore, during *Candida* infection, the regulation of JUNB may affect Treg cells in resisting *Candida* infection. The research of Rynes et al. found that ATF3 can maintain the homeostasis of the metabolism and immune system [40, 41]. The loss of ATF3 can cause chronic inflammation. Through NF-B/Relish in the ATF3 mutant, the overactive pro-inflammatory and stress signals caused by Jun N-terminal kinase and FOXO can remove the regulation of the important genes in immune defense [42]. In *Candida* infection, the main role of ATF3 is to inhibit inflammation. Studies have found that the adaptive immune response is regulated by EGR2 and EGR3 by uncoupling and expanding the time of T cell differentiation. EGR2 binds to and controls the expression of proliferation regulating genes (Myc and Myb), differentiation inhibitors (Bcl6, Id3) and inhibits transcription factors (Zeb2, RORa, RORc, and Bhlhe40) required for effector functions. EGR2 and EGR3 are upstream regulation factors of CD4 and CD8 T cells, which are essential for optimal response under limited immunopathology [43, 44]. In our research, EGR2 was a key adjustment factor. While its impact has been confirmed, the effect of EGR3 was not reflected clearly.

BTG2 is an archetype member of the BTG/Tob antiproliferative protein family, and its expression is related to various cellular processes, for instance, the generation cycle, divergence, or apoptosis of cells. BTG2 may act as a regulatory factor of the intracellular signal transduction cascade [45, 46]. BTG2 expression is induced through a p53-dependent mechanism, and the function of BTG2 may be related to cell cycle control and DNA damage reaction [47, 48].

Overall, bioinformatics analysis can be used to study the complex underlying molecular mechanisms related to diseases. In this study, the hub genes related to *Candida* infection were identified. However, further experimentation is required to verify these predicted results from bioinformatics analysis. This study has some limitations. Firstly, the number of sample of each group of *Candida* species was 18, therefore, the sample size of the study was relatively small. Secondly, the research sample did not eliminate factors like gender, whether the gene expression profiles were infected with other diseases, or whether drugs were used. This may have affected the factors of *Candida* infection in the gene expression.

## Supplementary Information

**Additional file 1: Table S1.** Significant enrichment of GO terms for *Candida albicans* (top 5 according to* P* value).

**Additional file 2: Table S2.** Significant enrichment of GO terms for *Candida glabrata* (top 5 according to* P* value).

**Additional file 3: Table S3.** Significant enrichment of GO terms for *Candida parapsilosis* (top 5 according to* P* value).

**Additional file 4: Table S4.** Significant enrichment of GO terms for *Candida tropicalis* (top 5 according to *P* value).

**Additional file 5: Table S5.** Top 10 significantly enriched KEGG pathways of *Candida albicans* (according to* P* value).

**Additional file 6: Table S6.** Top 10 significantly enriched KEGG pathways of *Candida glabrata* (according to* P* value).

**Additional file 7: Table S7.** Top 10 significantly enriched KEGG pathways of *Candida parapsilosis* (according to* P* value).

**Additional file 8: Table S8.** Top 10 significantly enriched KEGG pathways of *Candida tropicalis* (according to* P* value).

**Additional file 9: Figure S1. **Construction of the PPI network from four groups of *Candida*. *Candida albicans *(A), *Candida glabrata *(B), *Candida parapsilosis* (C) and *Candida tropicalis* (D).

**Additional file 10: Figure S2. **Top 10 hub genes of four groups of *Candida.* *Candida albicans* (A), *Candida glabrata *(B), *Candida parapsilosis *(C) and *Candida tropicalis *(D).

## Data Availability

The dataset(s) supporting the conclusions of this article is available in the Gene Expression Omnibus (GEO) database, http://www.ncbi.nlm.nih.gov/geo/.

## Abbreviations

GEO: Gene expression omnibus
DEGs: Differentially expressed genes
GO: Gene Ontology
KEGG: Kyoto Encyclopedia of Genes and Genomes
PPI: Protein–protein interaction
*C. albicans*: *Candida albicans*
*C. glabrata*: *Candida glabrata*
*C. tropicalis*: *Candida tropicalis*
*C. parapsilosis*: *Candida parapsilosis*
*C. krusei*: *Candida krusei*
NCBI: National Center for Biotechnology Information
MF: Molecular function
BP: Biological process
CC: Cellular component
MCC: Maximal Clique Centrality
PRRs: Pattern recognition receptors
PAMPs: Pathogen-associated molecular patterns
TLRs: Toll-like receptors
CLRs: C-type lectin receptors
NLRs: NOD-like receptors
RLRs: RIG-I-like receptors
ROS: Reactive oxygen species
DCs: Dendritic cells
TH: T helper
IL-17: Interleukin-17
IFNγ: Interferon-γ
TNF: Tumor necrosis factor
NEMO: NF-kappa B essential modulator
NIK: NF-κB-inducing kinase
