# Supplementary material for: Identification of differentially expressed genes and signaling pathways with Candida infection by bioinformatics analysis
Source: Eur J Med Res. 2022 Mar 21;27:43. doi: 10.1186/s40001-022-00651-w (PMC8935812; doi:10.1186/s40001-022-00651-w)
Supplement: Supplementary file 1 — Additional file 1: Table S1. Significant enrichment of GO terms for Candida albicans (top 5 according to P value). [file 40001_2022_651_MOESM1_ESM.docx]

Table S1 Significant enrichment of GO terms for *Candida albicans* (top 5 according to *P* value).

| Ontology | ID | Description | *P* value | Count | Gene name |
| --- | --- | --- | --- | --- | --- |
| BP | GO:0002521 | leukocyte differentiation | 4.64215E-06 | 13 | NFKBIZ/EGR1/EGR3/MERTK/JUNB/MMP9/CSF1R/BCL2/AGER/PRELID1/SLAMF8/TFRC/PTGER4 |
| BP | GO:0002694 | regulation of leukocyte activation | 5.42111E-06 | 13 | NFKBIZ/EGR3/MILR1/MERTK/VSIG4/BCL2/IRS2/AGER/PRELID1/PTAFR/SLAMF8/TFRC/MAP3K8 |
| BP | GO:0019083 | viral transcription | 8.91414E-06 | 8 | ZFP36/IFITM3/RPL23A/RPSA/RPL7A/RPS2/RPL6/CHD1 |
| BP | GO:0019080 | viral gene expression | 1.55155E-05 | 8 | ZFP36/IFITM3/RPL23A/RPSA/RPL7A/RPS2/RPL6/CHD1 |
| BP | GO:0000956 | nuclear-transcribed mRNA catabolic process | 1.94002E-05 | 8 | ZFP36/BTG2/RPL23A/RPSA/RPL7A/RPS2/TOB1/RPL6 |
| CC | GO:0022626 | cytosolic ribosome | 1.86055E-05 | 6 | DDX3X/RPL23A/RPSA/RPL7A/RPS2/RPL6 |
| CC | GO:0044445 | cytosolic part | 0.000200931 | 7 | DDX3X/RPL23A/NLRC4/RPSA/RPL7A/RPS2/RPL6 |
| CC | GO:1904724 | tertiary granule lumen | 0.000287344 | 4 | LRG1/MMP9/CXCL1/FOLR3 |
| CC | GO:0070820 | tertiary granule | 0.000382375 | 6 | LRG1/MMP9/PTAFR/SLC2A3/CXCL1/FOLR3 |
| CC | GO:0044391 | ribosomal subunit | 0.00050775 | 6 | DDX3X/RPL23A/RPSA/RPL7A/RPS2/RPL6 |
| MF | GO:0008329 | signaling pattern recognition receptor activity | 0.000127172 | 3 | MARCO/PTAFR/CLEC7A |
| MF | GO:0038187 | pattern recognition receptor activity | 0.000151954 | 3 | MARCO/PTAFR/CLEC7A |
| MF | GO:0071889 | 14-3-3 protein binding | 0.000718234 | 3 | ZFP36/IRS2/SIK1 |
| MF | GO:0038024 | cargo receptor activity | 0.001595392 | 4 | CD163/MARCO/AGER/TFRC |
| MF | GO:0004953 | icosanoid receptor activity | 0.002183586 | 2 | LTB4R/PTGER4 |

GO: gene ontology; BP: biological process; CC:cellular component; MF: molecular function.
