# Supplementary material for: Identification of differentially expressed genes and signaling pathways with Candida infection by bioinformatics analysis
Source: Eur J Med Res. 2022 Mar 21;27:43. doi: 10.1186/s40001-022-00651-w (PMC8935812; doi:10.1186/s40001-022-00651-w)
Supplement: Supplementary file 2 — Additional file 2: Table S2. Significant enrichment of GO terms for Candida glabrata (top 5 according to P value). [file 40001_2022_651_MOESM2_ESM.docx]

Table S2 Significant enrichment of GO terms for *Candida glabrata* (top 5 according to *P* value).

| Ontology | ID | Description | *P* value | Count | Gene name |
| --- | --- | --- | --- | --- | --- |
| BP | GO:0019083 | viral transcription | 1.55961E-08 | 11 | ZFP36/RPL23A/RPSA/RPL41/RPS26/RPL6/RPL7A/CCL3/RPL21/RPS2/CCL4 |
| BP | GO:0006413 | translational initiation | 2.20396E-08 | 11 | DDX3X/RPL23A/RPSA/RPL41/RPS26/RPL6/PPP1R15A/RPL7A/TNF/RPL21/RPS2 |
| BP | GO:0019080 | viral gene expression | 3.42813E-08 | 11 | ZFP36/RPL23A/RPSA/RPL41/RPS26/RPL6/RPL7A/CCL3/RPL21/RPS2/CCL4 |
| BP | GO:0070972 | protein localization to endoplasmic reticulum | 1.16805E-07 | 9 | RPL23A/RPSA/RPL41/RPS26/RPL6/PPP1R15A/RPL7A/RPL21/RPS2 |
| BP | GO:0006614 | SRP-dependent cotranslational protein targeting to membrane | 2.82212E-07 | 8 | RPL23A/RPSA/RPL41/RPS26/RPL6/RPL7A/RPL21/RPS2 |
| CC | GO:0022626 | cytosolic ribosome | 8.77636E-09 | 9 | DDX3X/RPL23A/RPSA/RPL41/RPS26/RPL6/RPL7A/RPL21/RPS2 |
| CC | GO:0044445 | cytosolic part | 8.06982E-08 | 11 | DDX3X/RPL23A/RPSA/RPL41/RPS26/NLRC4/RPL6/RPL7A/RPL21/RPS2/NLRP3 |
| CC | GO:0044391 | ribosomal subunit | 1.53373E-06 | 9 | DDX3X/RPL23A/RPSA/RPL41/RPS26/RPL6/RPL7A/RPL21/RPS2 |
| CC | GO:0022625 | cytosolic large ribosomal subunit | 1.89476E-05 | 5 | RPL23A/RPL41/RPL6/RPL7A/RPL21 |
| CC | GO:0005840 | ribosome | 3.19669E-05 | 9 | DDX3X/RPL23A/RPSA/RPL41/RPS26/RPL6/RPL7A/RPL21/RPS2 |
| MF | GO:0001228 | DNA-binding transcription activator activity, RNA polymerase II-specific | 0.000164252 | 10 | EGR2/FOSB/NR4A1/EGR1/NR4A2/JUNB/ATF3/IER2/CSRNP1/NR4A3 |
| MF | GO:0031726 | CCR1 chemokine receptor binding | 0.000381835 | 2 | CCL3/CCL4 |
| MF | GO:0003735 | structural constituent of ribosome | 0.000454066 | 6 | RPL23A/RPL41/RPL6/RPL7A/RPL21/RPS2 |
| MF | GO:0045296 | cadherin binding | 0.000548357 | 8 | DDX3X/RPL23A/ITGA6/RPS26/RPL6/RPL7A/TRIM25/RPS2 |
| MF | GO:0031730 | CCR5 chemokine receptor binding | 0.000795324 | 2 | CCL3/CCL4 |
