# Supplementary material for: Identification of differentially expressed genes and signaling pathways with Candida infection by bioinformatics analysis
Source: Eur J Med Res. 2022 Mar 21;27:43. doi: 10.1186/s40001-022-00651-w (PMC8935812; doi:10.1186/s40001-022-00651-w)
Supplement: Supplementary file 3 — Additional file 3: Table S3. Significant enrichment of GO terms for Candida parapsilosis (top 5 according to P value). [file 40001_2022_651_MOESM3_ESM.docx]

Table S3 Significant enrichment of GO terms for *Candida parapsilosis* (top 5 according to *P* value).

| Ontology | ID | Description | *P* value | Count | Gene name |
| --- | --- | --- | --- | --- | --- |
| BP | GO:0002521 | leukocyte differentiation | 4.43217E-13 | 23 | EGR3/EGR1/NFKBIZ/JUNB/MERTK/TNF/CCL3/AGER/CSF1R/NLRP3/CSF1/PTGER4/ZNF683/SRF/CD83/TRIB1/NFKBID/IL27/JUN/MMP9/IL6/VEGFA/TFRC |
| BP | GO:0002694 | regulation of leukocyte activation | 2.71033E-10 | 20 | EGR3/NFKBIZ/MERTK/VSIG4/MILR1/TNF/CCL3/MIR27A/AGER/NLRP3/NR4A3/ZNF683/MAP3K8/CD83/NFKBID/IL27/IL6/TFRC/HES1/IL1B |
| BP | GO:0042110 | T cell activation | 2.32671E-09 | 18 | EGR3/EGR1/NFKBIZ/VSIG4/MIR27A/AGER/NLRP3/PTGER4/ZNF683/SRF/MAP3K8/CD83/NFKBID/IL27/IL6/TFRC/HES1/IL1B |
| BP | GO:1903037 | regulation of leukocyte cell-cell adhesion | 2.779E-09 | 15 | EGR3/NFKBIZ/VSIG4/TNF/MIR27A/AGER/NLRP3/NR4A3/MAP3K8/CD83/NFKBID/IL6/TFRC/HES1/IL1B |
| BP | GO:1903039 | positive regulation of leukocyte cell-cell adhesion | 4.02755E-09 | 13 | EGR3/NFKBIZ/TNF/AGER/NLRP3/NR4A3/MAP3K8/CD83/NFKBID/IL6/TFRC/HES1/IL1B |
| CC | GO:1990682 | CSF1-CSF1R complex | 4.6599E-05 | 2 | CSF1R/CSF1 |
| CC | GO:0035976 | transcription factor AP-1 complex | 0.000459733 | 2 | JUNB/JUN |
| CC | GO:0022626 | cytosolic ribosome | 0.000494615 | 5 | RPSA/DDX3X/RPL21/RPL23A/RPL6 |
| CC | GO:1904724 | tertiary granule lumen | 0.000547651 | 4 | CXCL1/PTX3/FOLR3/MMP9 |
| CC | GO:0044445 | cytosolic part | 0.000565639 | 7 | RPSA/DDX3X/RPL21/RPL23A/NLRP3/RPL6/NLRC4 |
| MF | GO:0005125 | cytokine activity | 1.01221E-07 | 10 | OSM/CKLF/TNF/CCL3/CSF1/CCL4/IL27/IL6/VEGFA/IL1B |
| MF | GO:0005126 | cytokine receptor binding | 2.02703E-07 | 11 | OSM/CKLF/TNF/CCL3/ENG/CSF1/CCL4/IL27/IL6/VEGFA/IL1B |
| MF | GO:0001228 | DNA-binding transcription activator activity, RNA polymerase II-specific | 2.64802E-06 | 13 | EGR2/FOSB/NR4A1/EGR1/JUNB/NR4A2/ATF3/IER2/CSRNP1/NR4A3/SRF/FOSL2/JUN |
| MF | GO:0048018 | receptor ligand activity | 8.7245E-05 | 11 | OSM/CKLF/TNF/CCL3/CXCL1/CSF1/CCL4/IL27/IL6/VEGFA/IL1B |
| MF | GO:0030545 | receptor regulator activity | 0.000158846 | 11 | OSM/CKLF/TNF/CCL3/CXCL1/CSF1/CCL4/IL27/IL6/VEGFA/IL1B |
