# Supplementary material for: Identification of differentially expressed genes and signaling pathways with Candida infection by bioinformatics analysis
Source: Eur J Med Res. 2022 Mar 21;27:43. doi: 10.1186/s40001-022-00651-w (PMC8935812; doi:10.1186/s40001-022-00651-w)
Supplement: Supplementary file 4 — Additional file 4: Table S4. Significant enrichment of GO terms for Candida tropicalis (top 5 according to P value). [file 40001_2022_651_MOESM4_ESM.docx]

Table S4 Significant enrichment of GO terms for *Candida tropicalis* (top 5 according to *P* value).

| Ontology | ID | Description | P value | Count | Gene name |
| --- | --- | --- | --- | --- | --- |
| BP | GO:0002521 | leukocyte differentiation | 2.2269E-08 | 16 | NFKBIZ/EGR1/EGR3/MERTK/JUNB/CCL3/ZNF683/BCL2/TNF/NLRP3/PTGER4/TFRC/NFKBID/CSF1/LIF/FOS |
| BP | GO:0002694 | regulation of leukocyte activation | 1.77826E-07 | 15 | NFKBIZ/MILR1/EGR3/MERTK/CCL3/ZNF683/BCL2/TNF/NR4A3/MIR27A/NLRP3/MAP3K8/HES1/TFRC/NFKBID |
| BP | GO:1903706 | regulation of hemopoiesis | 5.9514E-07 | 14 | ZFP36/OSM/NFKBIZ/EGR3/CCL3/ZNF683/TNF/NR4A3/NLRP3/HES1/NFKBID/CSF1/LIF/FOS |
| BP | GO:0051384 | response to glucocorticoid | 1.58954E-06 | 8 | PTGS2/DUSP1/ZFP36/FOSB/BCL2/TNF/GJB2/FOS |
| BP | GO:0042110 | T cell activation | 1.92086E-06 | 13 | NFKBIZ/EGR1/EGR3/ZNF683/BCL2/CLEC7A/MIR27A/NLRP3/MAP3K8/HES1/PTGER4/TFRC/NFKBID |
| CC | GO:0035976 | transcription factor AP-1 complex | 1.76201E-06 | 3 | JUNB/DDIT3/FOS |
| CC | GO:0022626 | cytosolic ribosome | 1.67223E-05 | 6 | DDX3X/RPL23A/RPSA/RPL21/RPL6/RPS2 |
| CC | GO:1990622 | CHOP-ATF3 complex | 3.19049E-05 | 2 | ATF3/DDIT3 |
| CC | GO:0044445 | cytosolic part | 0.000178914 | 7 | DDX3X/RPL23A/RPSA/RPL21/RPL6/NLRP3/RPS2 |
| CC | GO:0044391 | ribosomal subunit | 0.000459581 | 6 | DDX3X/RPL23A/RPSA/RPL21/RPL6/RPS2 |
| MF | GO:0001228 | DNA-binding transcription activator activity, RNA polymerase II-specific | 3.32574E-07 | 13 | EGR2/FOSB/NR4A1/EGR1/NR4A2/JUNB/ATF3/CSRNP1/NR4A3/IER2/DDIT3/FOSL2/FOS |
| MF | GO:0008330 | protein tyrosine/threonine phosphatase activity | 0.000192602 | 2 | DUSP1/DUSP2 |
| MF | GO:0005125 | cytokine activity | 0.000253771 | 6 | OSM/CCL3/TNF/CCL4/CSF1/LIF |
| MF | GO:0031726 | CCR1 chemokine receptor binding | 0.000319805 | 2 | CCL3/CCL4 |
| MF | GO:0008140 | cAMP response element binding protein binding | 0.00047792 | 2 | DDIT3/SIK1 |
