# Supplementary material for: Identification of differentially expressed genes and signaling pathways with Candida infection by bioinformatics analysis
Source: Eur J Med Res. 2022 Mar 21;27:43. doi: 10.1186/s40001-022-00651-w (PMC8935812; doi:10.1186/s40001-022-00651-w)
Supplement: Supplementary file 5 — Additional file 5: Table S5. Top 10 significantly enriched KEGG pathways of Candida albicans (according to P value). [file 40001_2022_651_MOESM5_ESM.docx]

| ID | Description | *P* value | Gene name | Count |
| --- | --- | --- | --- | --- |
| hsa04668 | TNF signaling pathway | 0.000384408 | PTGS2/SOCS3/JUNB/MMP9/CXCL1/MAP3K8 | 6 |
| hsa04657 | IL-17 signaling pathway | 0.001189413 | PTGS2/FOSB/MMP9/CXCL8/CXCL1 | 5 |
| hsa04064 | NF-kappa B signaling pathway | 0.001795887 | PTGS2/TRIM25/BCL2/CXCL8/CXCL1 | 5 |
| hsa05161 | Hepatitis B | 0.002624062 | EGR2/EGR3/DDX3X/MMP9/BCL2/CXCL8 | 6 |
| hsa04380 | Osteoclast differentiation | 0.004803678 | FOSB/SOCS3/JUNB/CSF1R/FCGR1A | 5 |
| hsa05202 | Transcriptional misregulation in cancer | 0.005180068 | NFKBIZ/MMP9/CSF1R/JMJD1C/FCGR1A/CXCL8 | 6 |
| hsa05132 | Salmonella infection | 0.005344491 | NLRC4/CXCL8/CCL3L3/CXCL1 | 4 |
| hsa03010 | Ribosome | 0.010072098 | RPL23A/RPSA/RPL7A/RPS2/RPL6 | 5 |
| hsa04933 | AGE-RAGE signaling pathway in diabetic complications | 0.010675869 | EGR1/BCL2/AGER/CXCL8 | 4 |
| hsa04061 | Viral protein interaction with cytokine and cytokine receptor | 0.010675869 | CSF1R/CXCL8/CCL3L3/CXCL1 | 4 |

Table S5 Top 10 significantly enriched KEGG pathways of *Candida albicans* ( according to *P* value).
