# Supplementary material for: Identification of differentially expressed genes and signaling pathways with Candida infection by bioinformatics analysis
Source: Eur J Med Res. 2022 Mar 21;27:43. doi: 10.1186/s40001-022-00651-w (PMC8935812; doi:10.1186/s40001-022-00651-w)
Supplement: Supplementary file 6 — Additional file 6: Table S6. Top 10 significantly enriched KEGG pathways of Candida glabrata (according to P value). [file 40001_2022_651_MOESM6_ESM.docx]

Table S6 Top 10 significantly enriched KEGG pathways of *Candida glabrata* ( according to *P* value).

| ID | Description | p value | Count | Gene name |
| --- | --- | --- | --- | --- |
| hsa04668 | TNF signaling pathway | 4.17166E-07 | 9 | PTGS2/SOCS3/JUNB/TNF/FAS/CXCL1/MMP9/CSF1/MAP3K8 |
| hsa04061 | Viral protein interaction with cytokine and cytokine receptor | 1.99424E-06 | 8 | CSF1R/CCL3L1/TNF/CCL3/CXCL1/CSF1/CCL4L2/CCL4 |
| hsa04064 | NF-kappa B signaling pathway | 2.50481E-05 | 7 | PTGS2/TRIM25/TNF/CXCL1/GADD45B/CCL4L2/CCL4 |
| hsa05132 | Salmonella infection | 6.88776E-05 | 6 | CCL3L1/NLRC4/CCL3/CXCL1/CCL4L2/CCL4 |
| hsa04060 | Cytokine-cytokine receptor interaction | 0.000190467 | 10 | OSM/CSF1R/CCL3L1/TNF/CCL3/FAS/CXCL1/CSF1/CCL4L2/CCL4 |
| hsa04620 | Toll-like receptor signaling pathway | 0.000257315 | 6 | CCL3L1/TNF/CCL3/CCL4L2/MAP3K8/CCL4 |
| hsa04625 | C-type lectin receptor signaling pathway | 0.000257315 | 6 | EGR2/PTGS2/EGR3/TNF/NLRP3/CLEC7A |
| hsa04380 | Osteoclast differentiation | 0.000782445 | 6 | FOSB/SOCS3/JUNB/CSF1R/TNF/CSF1 |
| hsa04010 | MAPK signaling pathway | 0.00089945 | 9 | NR4A1/DUSP1/RAPGEF2/CSF1R/TNF/FAS/CSF1/GADD45B/MAP3K8 |
