# Supplementary material for: Identification of differentially expressed genes and signaling pathways with Candida infection by bioinformatics analysis
Source: Eur J Med Res. 2022 Mar 21;27:43. doi: 10.1186/s40001-022-00651-w (PMC8935812; doi:10.1186/s40001-022-00651-w)
Supplement: Supplementary file 7 — Additional file 7: Table S7. Top 10 significantly enriched KEGG pathways of Candida parapsilosis (according to P value). [file 40001_2022_651_MOESM7_ESM.docx]

Table S7 Top 10 significantly enriched KEGG pathways of *Candida parapsilosis* ( according to *P* value).

| ID | Description | *P* value | Count | Gene name |
| --- | --- | --- | --- | --- |
| hsa04668 | TNF signaling pathway | 1.15798E-09 | 12 | PTGS2/SOCS3/JUNB/TNF/CXCL1/CSF1/MAP3K8/JUN/MMP9/CXCL2/IL6/IL1B |
| hsa05132 | Salmonella infection | 8.73047E-09 | 10 | CCL3L1/CCL3/CXCL1/CCL4L2/CCL4/NLRC4/JUN/CXCL2/IL6/IL1B |
| hsa05323 | Rheumatoid arthritis | 3.0109E-08 | 10 | CCL3L1/TNF/CCL3/CXCL1/CSF1/JUN/CXCL2/IL6/VEGFA/IL1B |
| hsa04061 | Viral protein interaction with cytokine and cytokine receptor | 6.08842E-08 | 10 | CCL3L1/TNF/CCL3/CXCL1/CSF1R/CCL4L2/CSF1/CCL4/CXCL2/IL6 |
| hsa04064 | NF-kappa B signaling pathway | 7.36919E-08 | 10 | PTGS2/TNF/CXCL1/GADD45B/TRIM25/CCL4L2/CCL4/BCL2A1/CXCL2/IL1B |
| hsa04657 | IL-17 signaling pathway | 3.87977E-07 | 9 | PTGS2/FOSB/TNF/CXCL1/JUN/MMP9/CXCL2/IL6/IL1B |
| hsa04620 | Toll-like receptor signaling pathway | 1.01176E-06 | 9 | CCL3L1/TNF/CCL3/CCL4L2/CCL4/MAP3K8/JUN/IL6/IL1B |
| hsa04380 | Osteoclast differentiation | 5.75418E-06 | 9 | FOSB/SOCS3/JUNB/TNF/CSF1R/CSF1/FOSL2/JUN/IL1B |
| hsa04060 | Cytokine-cytokine receptor interaction | 7.9376E-06 | 13 | OSM/CCL3L1/TNF/CCL3/CXCL1/CSF1R/CCL4L2/CSF1/CCL4/IL27/CXCL2/IL6/IL1B |
| hsa04010 | MAPK signaling pathway | 8.23559E-06 | 13 | NR4A1/DUSP1/TNF/RAPGEF2/GADD45B/CSF1R/CSF1/SRF/MAP3K8/JUN/DUSP2/VEGFA/IL1B |
