# Supplementary material for: Identification of differentially expressed genes and signaling pathways with Candida infection by bioinformatics analysis
Source: Eur J Med Res. 2022 Mar 21;27:43. doi: 10.1186/s40001-022-00651-w (PMC8935812; doi:10.1186/s40001-022-00651-w)
Supplement: Supplementary file 8 — Additional file 8: Table S8. Top 10 significantly enriched KEGG pathways of Candida tropicalis (according to P value). [file 40001_2022_651_MOESM8_ESM.docx]

Table S8 Top 10 significantly enriched KEGG pathways of *Candida tropicalis* ( according to *P* value).

| ID | Description | p value | Count | Gene name |
| --- | --- | --- | --- | --- |
| hsa04064 | NF-kappa B signaling pathway | 2.11764E-07 | 9 | PTGS2/TRIM25/BCL2/CXCL1/TNF/GADD45B/CCL4L2/BCL2A1/CCL4 |
| hsa04668 | TNF signaling pathway | 4.74098E-07 | 9 | PTGS2/SOCS3/JUNB/CXCL1/TNF/MAP3K8/CSF1/LIF/FOS |
| hsa04061 | Viral protein interaction with cytokine and cytokine receptor | 2.42423E-05 | 7 | CCL3L1/CCL3/CXCL1/TNF/CCL4L2/CCL4/CSF1 |
| hsa04620 | Toll-like receptor signaling pathway | 3.12948E-05 | 7 | CCL3L1/CCL3/TNF/CCL4L2/MAP3K8/CCL4/FOS |
| hsa05132 | Salmonella infection | 7.48165E-05 | 6 | CCL3L1/CCL3/CXCL1/CCL4L2/CCL4/FOS |
| hsa04380 | Osteoclast differentiation | 0.000117984 | 7 | SOCS3/FOSB/JUNB/TNF/CSF1/FOSL2/FOS |
| hsa05323 | Rheumatoid arthritis | 0.000151036 | 6 | CCL3L1/CCL3/CXCL1/TNF/CSF1/FOS |
| hsa04210 | Apoptosis | 0.000172375 | 7 | PMAIP1/BCL2/TNF/GADD45B/BCL2A1/DDIT3/FOS |
| hsa04010 | MAPK signaling pathway | 0.000221559 | 10 | DUSP1/NR4A1/RAPGEF2/TNF/GADD45B/MAP3K8/DDIT3/CSF1/DUSP2/FOS |
| hsa04625 | C-type lectin receptor signaling pathway | 0.000278841 | 6 | EGR2/PTGS2/EGR3/CLEC7A/TNF/NLRP3 |
