# Supplementary material for: Identification of differentially expressed genes and signaling pathways with Candida infection by bioinformatics analysis
Source: Eur J Med Res. 2022 Mar 21;27:43. doi: 10.1186/s40001-022-00651-w (PMC8935812; doi:10.1186/s40001-022-00651-w)
Supplement: Supplementary file 9 — Additional file 9: Figure S1. Construction of the PPI network from four groups of Candida. Candida albicans (A), Candida glabrata (B), Candida parapsilosis (C) and Candida tropicalis (D). [file 40001_2022_651_MOESM9_ESM.pdf]

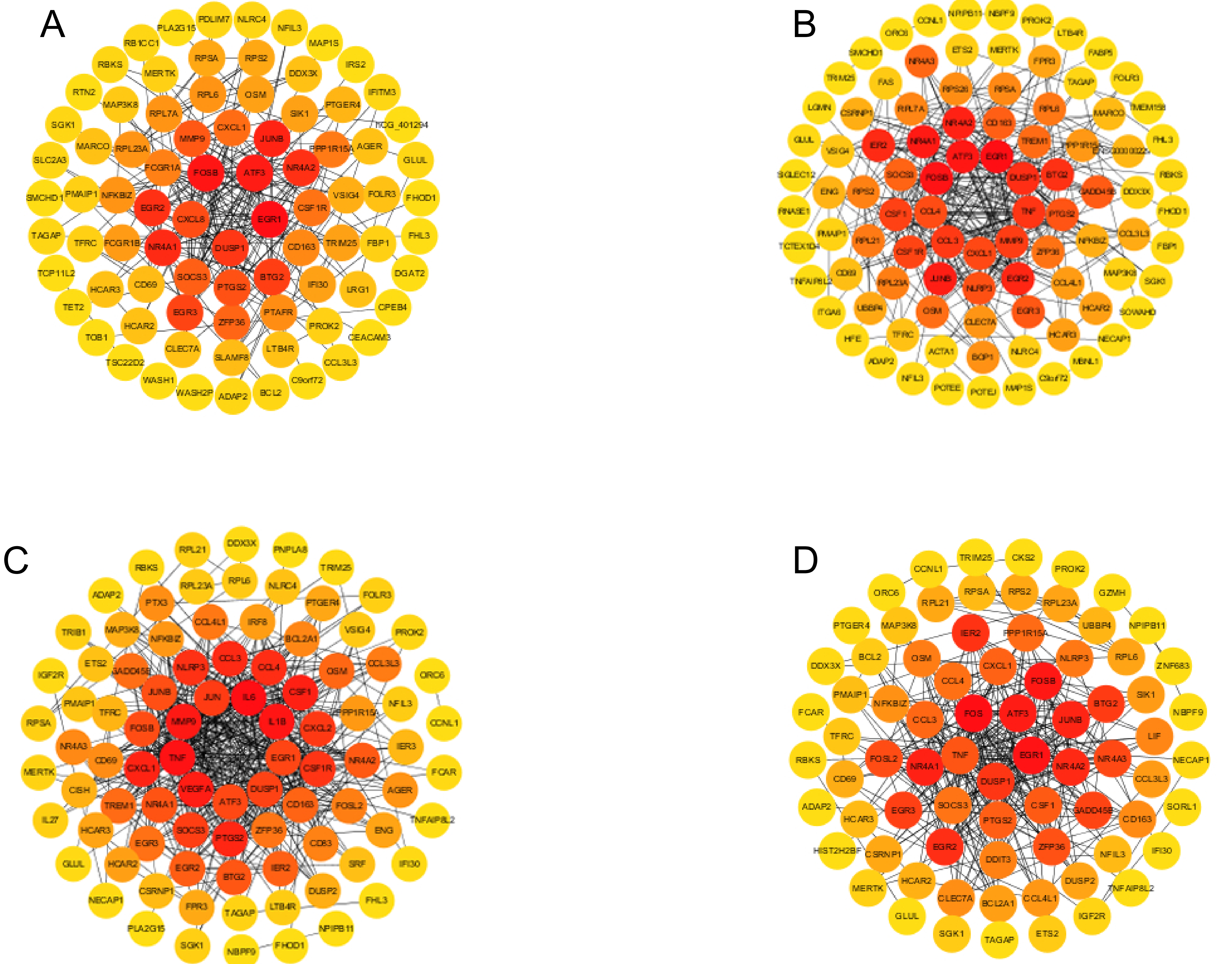
A
PLA2G15
PDLIM7
NLRC4
NFIL3
MAP1S
RB1CC1
RBKS
MERTK
RPSA
RPS2
DDX3X
IRS2
RTN2
RPL6
OSM
IFITM3
MAP3K8
RPL7A
SIK1
PTGER4
SGK1
CXCL1
LOC_401294
MARCO
RPL23A
MMP9
JUNB
PPP1R15A
AGER
SLC2A3
FCGR1A
FOSB
ATF3
NR4A2
GLUL
SMCHD1
PMAIP1
NFKBIZ
VSIG4
FOLR3
FHOD1
EGR2
CXCL8
CSF1R
EGR1
TAGAP
TFRC
FCGR1B
NR4A1
DUSP1
CD163
TRIM25
FBP1
FHL3
TCP11L2
SOCS3
BTG2
DGAT2
HCAR3
CD69
PTGS2
IFI30
LRG1
TET2
EGR3
PTAFR
CPEB4
HCAR2
ZFP36
PROK2
TOB1
CLEC7A
LTB4R
CEACAM3
SLAMF8
CCL3L3
TSC22D2
WASH1
C9orf72
WASH2P
ADAP2
BCL2
B
CCNL1
NFIL3
POTEE
POTEJ
MAP1S
C9orf72
C
RPL21
DDX3X
PNPLA8
RBKS
RPL6
TRIM25
RPL23A
NLRC4
ADAP2
PTX3
PTGER4
FOLR3
CCL4L1
IRF8
TRIB1
MAP3K8
VSIG4
PROK2
NFKBIZ
BCL2A1
ETS2
CCL3
CCL3L3
ORC6
IGF2R
NLRP3
CCL4
GADD45B
OSM
JUNB
JUN
IL6
CSF1
PMAIP1
NFIL3
TFRC
PPP1R15A
RPSA
MMP9
IL1B
CXCL2
CCNL1
FOSB
NR4A3
IER3
EGR1
TNF
CD69
NR4A2
CSF1R
MERTK
CXCL1
FCAR
VEGFA
DUSP1
CISH
ATF3
CD163
AGER
TREM1
NR4A1
FOSL2
TNFAIP8L2
IL27
SOCS3
ZFP36
HCAR3
ENG
PTGS2
CD83
EGR3
GLUL
HCAR2
IER2
IFI30
EGR2
SRF
BTG2
NECAP1
CSRNP1
DUSP2
FHL3
TAGAP
FPR3
LTB4R
NPIPB11
PLA2G15
SGK1
FHOD1
NBPF9
D
TRIM25
CKS2
CCNL1
PROK2
RPSA
RPS2
ORC6
RPL21
GZMH
RPL23A
IER2
PTGER4
UBBP4
MAP3K8
NPIPB11
PPP1R15A
OSM
BCL2
NLRP3
ZNF683
DDX3X
CXCL1
RPL6
CCL4
FOSB
SIK1
PMAIP1
BTG2
NFKBIZ
NBPF9
FCAR
CCL3
FOS
ATF3
JUNB
TFRC
LIF
RBKS
FOSL2
TNF
EGR1
NR4A3
NECAP1
NR4A1
NR4A2
CD69
DUSP1
CCL3L3
ADAP2
SOCS3
CSF1
HCAR3
GADD45B
EGR3
SORL1
PTGS2
CD163
EGR2
ZFP36
HIST2H2BF
IFI30
CSRNP1
NFIL3
DDIT3
DUSP2
MERTK
HCAR2
CLEC7A
TNFAIP8L2
BCL2A1
CCL4L1
GLUL
IGF2R
SGK1
TAGAP
ETS2
