# Supplementary figures and images for: Identification of differentially expressed genes and signaling pathways with Candida infection by bioinformatics analysis
Source: Eur J Med Res. 2022 Mar 21;27:43. doi: 10.1186/s40001-022-00651-w (PMC8935812; doi:10.1186/s40001-022-00651-w)

A

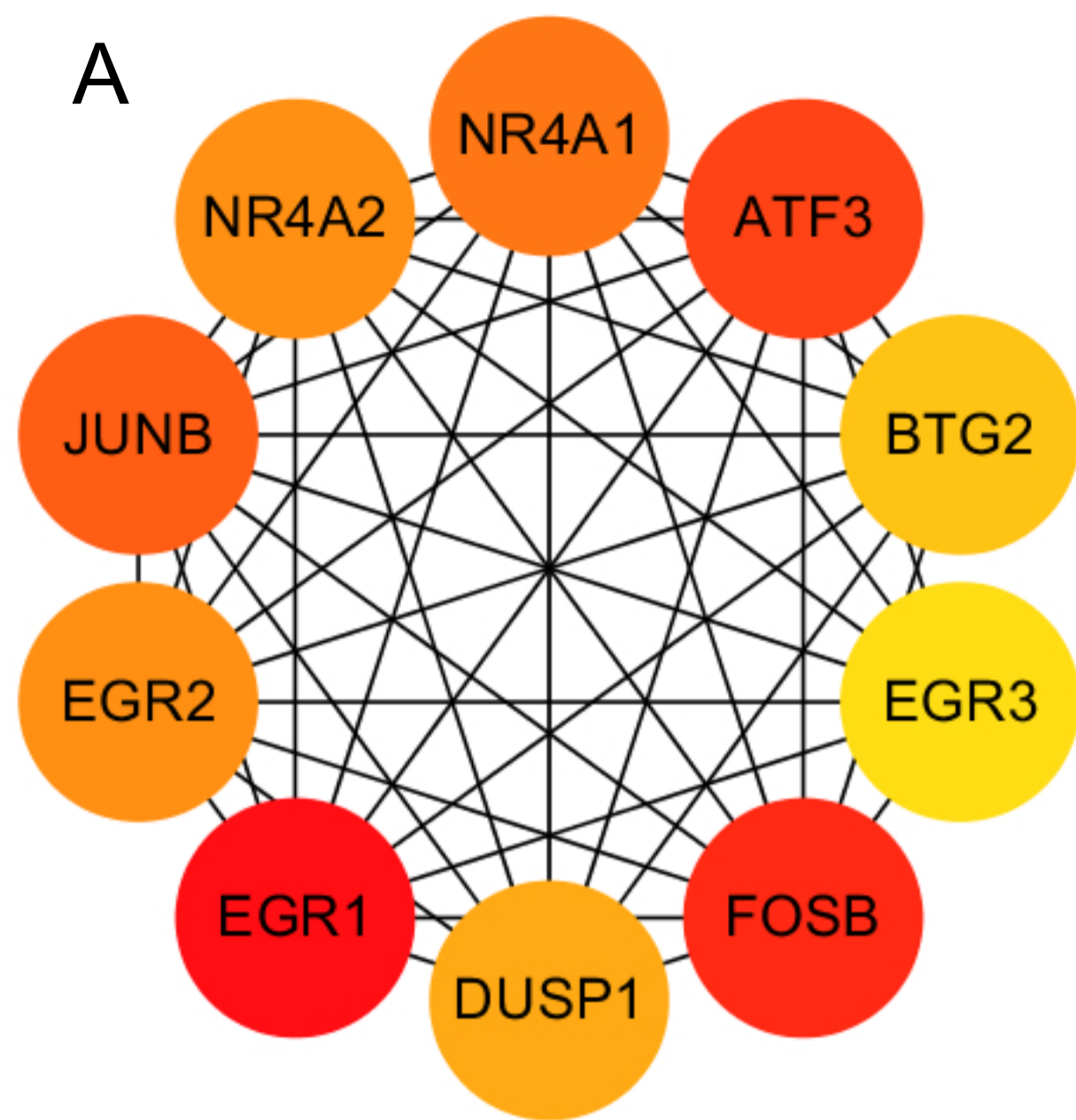

B

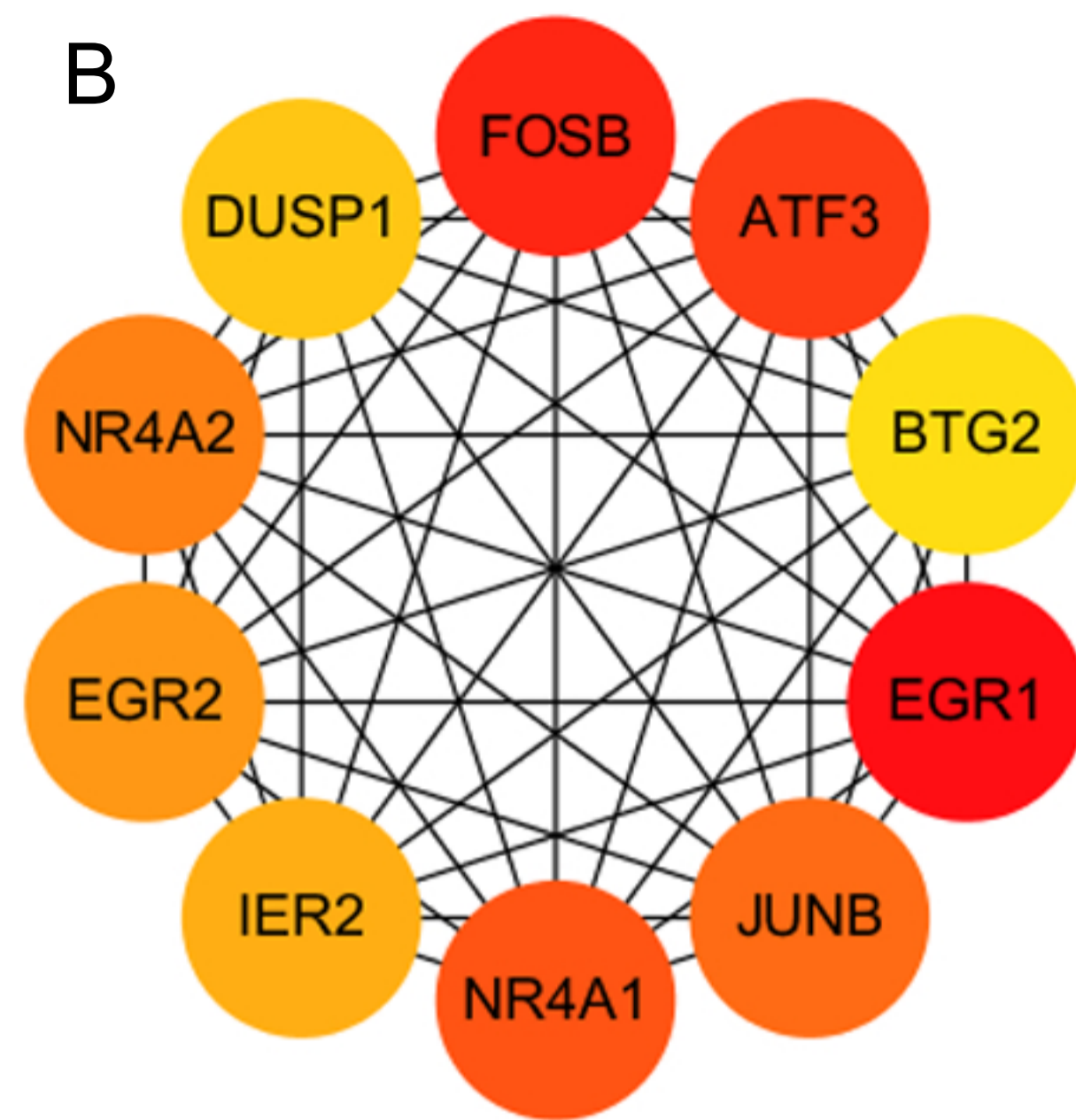

C

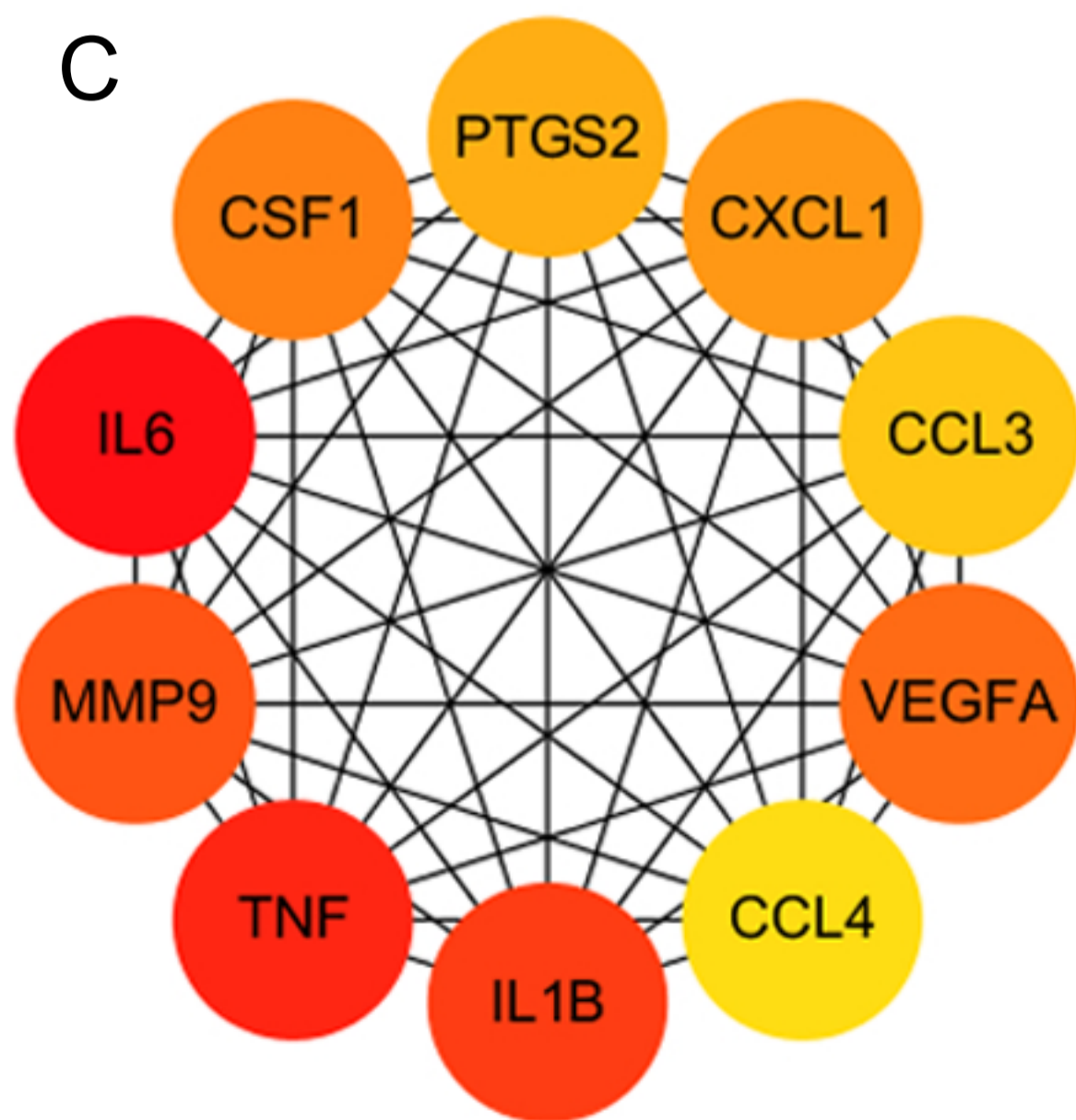

D

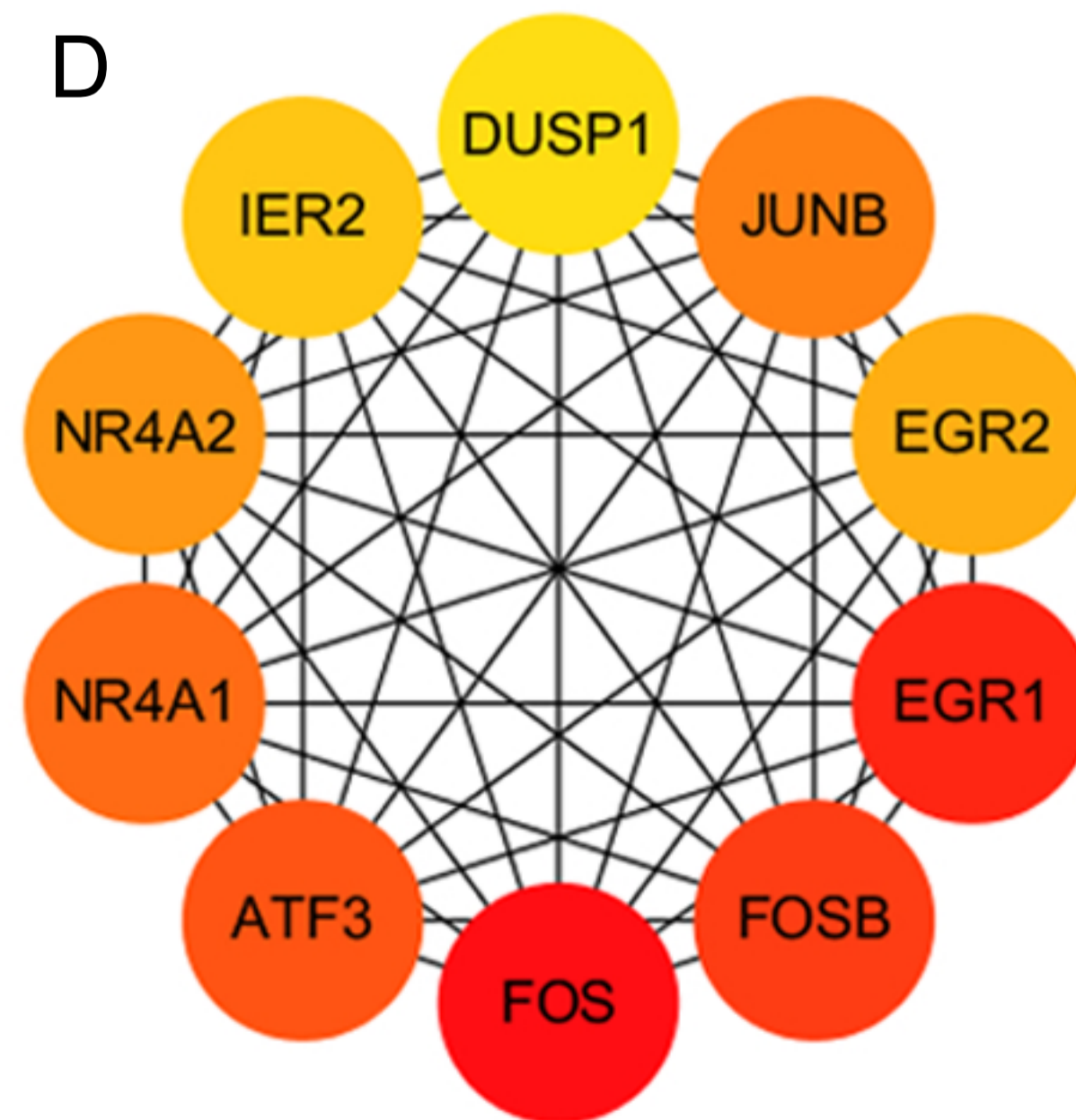

Supplement: Supplementary file 10 — Additional file 10: Figure S2. Top 10 hub genes of four groups of Candida. Candida albicans (A), Candida glabrata (B), Candida parapsilosis (C) and Candida tropicalis (D). [file 40001_2022_651_MOESM10_ESM.pdf]
